# What COVID-19 may teach us about interdisciplinarity

**DOI:** 10.1136/bmjgh-2020-004375

**Published:** 2020-12-15

**Authors:** Annemarie Mol, Anita Hardon

**Affiliations:** Anthropology, Amsterdam Institute for Social Science Research, Amsterdam, Netherlands

**Keywords:** COVID-19, vaccines, public health, health systems

**Summary box**

- Policy-makers have trouble dealing with the diverging suggestions of different scientific disciplines as there are not always easy to align.
- Different disciplines operationalise COVID-19 in different ways, propose diverging interventions, and, added to that, also use contrasting parameters of success.
- Interdisciplinarity should not be treated as a matter of adding the pieces of a puzzle together, but rather as a mediation process in which no discipline has to submit to either object definitions or criteria for good research of any other.
- Policy-makers, funders and research institutions should foster diversity in academic ecosystems just as is the case for biological ecosystems.
- Researchers need to attune to each other’s research styles as they work together to tackle the diverse aspects of the current pandemic in a science-based way.

Interdisciplinarity is often cast as a matter of different disciplines looking at a shared object from different perspectives such that each discipline highlights a different aspect of that object. The task at hand then consists in putting pieces of a puzzle together so as to make apparent the entire picture, see figure 1.

**Figure 1:**
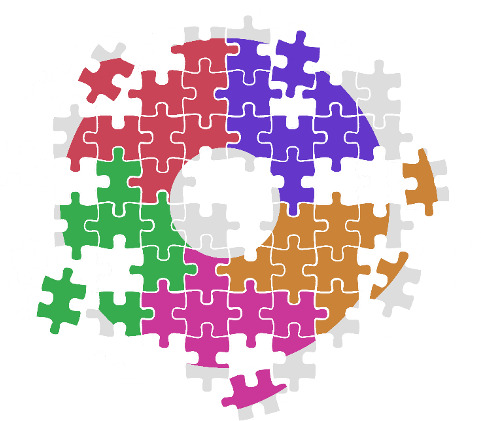
Interdisciplinarity imagined as combining pieces of a puzzle.

However, when different disciplines congregate, the sum total is often not an easily assembled coherent picture. On the contrary, the various conclusions reached by different disciplines may well point in different directions. Visual metaphors like ‘perspectives’ and ‘pieces of a puzzle’ do not aid the understanding of the tensions and clashes that interdisciplinarity tends to involve. In this article, we, therefore, propose a different epistemological take on interdisciplinarity. Building on decades of research into medical practices, we use COVID-19 as an example to argue that more useful than the above metaphors is stating that different disciplines handle reality in different ways.^1–3^ They draw on different techniques, address different concerns and operationalise their object of inquiry in different ways. They foster different paradigms. This means that even when, say, virologists, clinicians, physicists, epidemiologists, immunologists, economists and sociologists all use the term ‘COVID-19’ what they actually grapple with is not the same entity. Indeed, quite like the proverbial apples and oranges, the various entities that researchers working in different disciplines study cannot simply be added together to create a meaningful whole.

Thus, interdisciplinarity does not accord with the metaphor of the jigsaw puzzle in which each discipline adds a piece until ‘the whole picture’ is laid out on the table. Instead, moving from a visual metaphor to an analytics oriented on practice, we suggest that different disciplines engage with reality each in their own way. These ways are not closed off to one another. Different disciplines readily draw on each other’s work and their practitioners may collaborate. However, it also happens that they pull and push in different directions. Hence, the tensions and clashes.

What follows from this epistemological reorientation is that good interdisciplinarity is not simply a matter of achieving completeness. Rather, it requires paying attention to the diverse concerns of different disciplines and incorporating responsive negotiation of their collaborative possibilities and the tensions between them.^4^ As part of this endeavour, it is crucial to achieve lucid insight into the ways in which different disciplines (or, for that matter, sub-disciplines and sub-sub-disciplines) operationalise their object of inquiry and, each in their own way, respond to the concerns they share, see figure 2.

**Figure 2:**
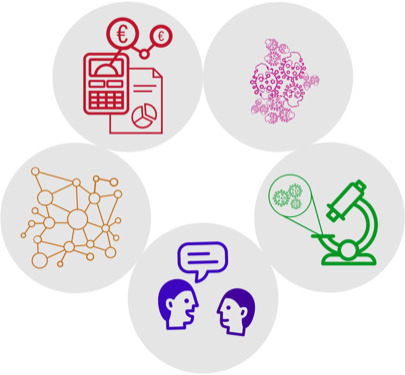
Different ways of operationalising COVID-19.

We support this argument with an outline of what the coexistence of disciplinary paradigms amounts to in the case of COVID-19. To be sure, the extensive body of research already conducted on COVID-19 cannot be outlined in just a few pages, so ours is not a mapping exercise. Instead, we employ exemplary simplifications to illustrate a shift in understandings of interdisciplinarity. We propose no longer approaching interdisciplinarity as a matter of fitting together complementary aspects of an object, but rather as negotiating the juxtaposition of its potentially contrasting versions.

## Versions of COVID-19

The disciplines of virology and clinical medicine collaborate closely. For instance, the diagnostic tests that allow clinicians to ascertain if a patient is indeed infected with SARS-CoV-2 were crafted by virologists, while virologists learn from clinicians how the virus impacts its human hosts.^5^ At the same time, the object of these two disciplines is different. Virologists study all kinds of viruses, and their objects of inquiry include viruses’ genes, history, hosts, levels of virulence and transmission routes. This means that, for virologists, ‘COVID-19 is a contagious disease due to an infection by a specific type of coronavirus: SARS-CoV-2’.^6^ In the world of virologists, the surge of COVID-19, while new in its particulars, was an accident waiting to happen. They had been warning us for decades that serious pandemics were going to arise from virus jumping from animal to human populations. The starting point for clinicians is not the SARS-CoV-2 virus, but the ways it affects its human hosts. For clinicians, COVID-19 is a disease that causes havoc in the bodies of unfortunate patients. This turned out to be full of surprises.^7^ The particular assaults on lung capacities and blood clotting mechanisms that clinicians observed in their patients with COVID-19 were new. Existing interventions were adapted, pathophysiological pathways unravelled. Hence, while there are marked crossovers between pursuing a virus and treating a patient’s body, the precise objects that these two disciplines operationalise are not the same.

While clinicians were trying to save their patients’ lives, outside hospitals, preventive measures were implemented. These were oriented around blocking the most probable routes the virus might take from one host to another. People were warned to wash their hands, cough into their elbows and avoid handshakes. They were asked to maintain a significant distance between their bodies. In this context, physicists of fluids started to wonder what distance might be significant enough, given the behaviour of fluids. They operationalised COVID-19 as a disease caused by a virus hitchhiking from one body to the next, dissolved in bodily fluids, and set up laboratory experiments to discover how far bodily fluids travel. Volunteers were asked to breath, cough or sneeze and the researchers rendered the resulting droplets visual.^8^ These experiments showed that the emitted droplets, both large and small, cluster together and are propelled forward in clouds. This means that their reach is a lot farther than the 1–2 m distance recommended in public health advisories.

Most biomedical researchers were not impressed. For even in the absence of preventive measures, most people with COVID-19 were less contagious than, say, people who have measles, known to be transmitted by small droplets. The physicists might have shown that, under experimental conditions, aerosols may possibly travel from one body to another, but the biomedical experts wanted to know the probability of this transmission route resulting in people becoming infected with SARS-CoV-2. They did not envision individuals, but populations. Hence, they turned to infectious disease epidemiology, the discipline for which COVID-19 is a contagious viral disease spreading in its own specific ways through human populations. In epidemiology, the transmission of the virus is not experimentally orchestrated, but painstakingly counted and traced in real-life situations.^9^ The data points that allow for the counting depend on the availability of test kits and laboratory supplies, skilled personnel and the readiness of people to undergo testing. One dire problem has been how to count those who have no or only mild symptoms.

Then, there is the tracing, which requires detective work. Is the outbreak among the members of a choir due to their singing, or does a thorough investigation reveal that all those infected gathered together in a small corridor for coffee after rehearsal?

Possible, probable and actual transmission routes are different phenomena and hence different objects to research. Even so, they share something in common: they are all transmission routes. Studying them helps to shape the hygienic measures that seek to prevent the virus from reaching its human hosts. Researchers taking their cues from immunology do not invest in transmission routes, but foreground the hosts.^10^ For them, COVID-19 is an infectious disease that stimulates immune systems in intriguingly diverse ways. They underline that some people, once infected, are affected far less than others, and wonder to which extent this the preparedness of their immune system may be involved in this. It might seem as if this question is adjacent to questions about transmission and only becomes relevant once hygiene has failed. But it is not that simple.^11^

The complexity becomes apparent in evaluations of the population-wide use of face masks. Within the logic of hygiene, face masks are meant to prevent the transmission of virus. Accordingly, evaluating their efficacy is a matter of comparing the number of positive tests between regions where face masks are worn and regions where they are not.^12^ Within an immunological logic, by contrast, the most significant characteristic of face masks is not necessarily that they block the transmission of the virus, but that they might lower the transmitted dose. This would be interesting if a small dose of SARS-CoV-2 were to trigger an immune response without developing into a full-blown disease. In that case, small doses could serve as inoculations—at least for some people and for some time.^13^ The parameter of success for this particular use of face masks would not be fewer positive tests; there might even be more positive tests than in populations not wearing face masks. The parameter of success, in this case, would be the elicitation of a protective immune response, resulting in fewer cases of severe disease and fewer deaths.

The clash that so far has gained most public attention is that between the hygienic blocking of viral flows by means of lockdowns and the hampering of economic flows that results from them.^14^ If lockdowns are imposed, people—insofar as they have access to food and their housing is adequate—are supposed to come out on the other end intact and healthy. Blocking economic flows, by contrast, is not similarly reversible, as it creates a downward spiral. Once businesses have gone bankrupt and jobs are lost, it is not obvious how to revitalise economies. For economics, COVID-19 is a threat to the economy as measures to block flows of the virus also block monetary flows. This discipline does not disaggregate biological events from societal responses to it, as these jointly affect the economy. Moreover, the threat COVID-19 poses is worse in societies without a properly functioning welfare state, for when people without jobs have no money to spend, they cannot, once a lockdown ends, pay for the goods and services that might allow others to resume working and once more earn their keep.

However, while this vicious circle is often flagged as a straightforward argument against lockdowns, things are—again—not so simple. Hygiene and economics have different unities of calculation, which are graphed along different x-axes and y-axes, with recommendations that point in different directions—however, instead of simply clashing, they are also interdependent. After all, when too many people become seriously ill or even die, this, too, disrupts the social fabric and pushes the economy into decline. Hence, just as successful lockdowns depend on people having sufficient food and appropriate housing, a vigorous economy depends on a sufficiently healthy population.

One last example. As it is, most epidemiologists attempt to comprehend the pandemic by developing explanatory models. Using as their input all kinds of data that impress them as suitable, they hope to model the most likely routes according to which COVID-19 spreads through human populations.^1^ Above, we signalled that this probabilistic approach is in tension with the studies into possible transmission routes that physicists conduct. Here, we want to make a further point. Similarly to research by physicists, epidemiological models aim for generality. These models are abstracted from single instances, so that they may travel unhindered around the globe. This approach conflicts with the investment in specificities current in anthropology and other social sciences, where ‘human populations’ stand out as an undue abstraction. Which populations, when, where and under which circumstances?^16 17^ While models envision a generalised human, for social scientists, COVID-19 is a multifaceted problem faced by particular people, living under specific social and material conditions. This means that, even if, thanks to WHO coordination, COVID-19 has the same name across the globe, it is not the same reality everywhere.^18^ Living with this disease is a different plight in Brazil, Germany, China, Uganda, the USA, the Czech Republic, the Netherlands, Bangladesh and so on—and in each of these countries it is different for those with and without steady incomes, housing, gardens, parks, families or what have you.^19^

Social scientists share this investment in specificity with clinicians, who likewise take heed of the specificities of this singular patient in the here and now. But while clinicians in intensive care units prioritise such variables as oxygen saturation and blood levels of bradykinin, social scientists investigate such issues as overburdening due to double duties, suffering from loneliness, increases in domestic violence and deepening inequalities due to lack of schooling.^20^ The list is open ended, see box 1.

Box 1 **Multiple versions of COVID-19**

- Virologists: COVID-19: a contagious disease due to an infection by a specific type of coronavirus: SARS-CoV-2.
- Clinicians: COVID-19 is a disease that causes havoc in the bodies of unfortunate patients.
- Physicist: COVID-19 is caused by a virus hitchhiking from one body to the next, dissolved in bodily fluids.
- Epidemiologists: COVID-19 is a viral disease spreading through human populations in particular ways.
- Immunologists: COVID-19 is an infectious disease that stimulates human immune systems in intriguingly diverse ways.
- Economics: COVID-19 is a threat to the economy, since measures to block viral flows also block monetary flows.
- Anthropology: COVID-19 is a multifaceted problem faced by particular people, living under specific social and material conditions.

## Conclusion

The above examples clearly illustrate that interdisciplinarity, if it is to prove worthwhile, is not a matter of addition, but of negotiation. It thrives on attention to the concerns raised by each and every relevant discipline. It asks for a deft way of handling the tensions between possible interventions, however much they diverge. For this much is obvious: Given the diversity of scientific repertoires, the expectation that ‘science’, in the singular, might eventually, once it has put all the pieces of the puzzle together, make sense of COVID-19 ‘as a whole’ and then generate univocal recommendations is bound to end in disappointment. While it is crucial that policymakers avoid politicisation of the pandemic, and instead heed insights from the sciences, this is truly difficult as the various suggestions that different scientific disciplines provide are not always easy to align. Here lies a task for academics. We would do well to tackle the complexities of our disunity head on. Instead of dreaming about ‘complete pictures’, we should engage in interdisciplinary conversations, realising that collaborating requires attention to equivocations, crafting compromises and wondering how and where it might be possible to accommodate divergent goals.

In the long run, this means that different research styles all deserve to be accorded space to continue along their own paths, without having to submit either to the object definitions or the criteria for good research of any other discipline. It means that diversity deserves to be fostered in academic ecosystems just as much as it does in biological ecosystems. It means that the virtues of inquisitiveness, tenaciousness and modesty should be advocated simultaneously. The coexistence in difference that we argue for is not served by discussions that comply with the formats for legal disputes or debating competitions, destined to end with a single winner. What is required, instead are conversations that take their inspiration from a democratic respect for minorities and from mediation, where ongoing differences are taken for granted, while solutions are sought that aim to do justice to each interlocutor’s particular intellectual and practical stakes. Our collective response to the current COVID-19 pandemic might have been more efficacious had we worked in this way. Well after our present predicament is in the past, the ideal of coexisting in difference is bound to allow for creative and generative kinds of interdisciplinarity.

## References

[1] Mol A The body multiple: ontology in medical practice. Durham, NC: Duke University Press, 2002.

[2] Hardon A, Pool R Anthropologists in global health experiments. Med Anthropol 2016;35:447–51. 10.1080/01459740.2016.1177046 27618222

[3] Mol A Living with diabetes: care beyond choice and control. Lancet 2009;373:1756–7. 10.1016/S0140-6736(09)60971-5 19480076

[4] Moreira T Heterogeneity and coordination of blood pressure in neurosurgery. Soc Stud Sci 2006;36:69–97. 10.1177/0306312705053051

[5] Corman VM, Landt O, Kaiser M, et al Detection of 2019 novel coronavirus (2019-nCoV) by real-time RT-PCR. Eurosurveillance 2020;25 10.2807/1560-7917.ES.2020.25.3.2000045 PMC6988269 31992387

[6] Andersen KG, Rambaut A, Lipkin WI, et al The proximal origin of SARS-CoV-2. Nat Med 2020;26:450–2. 10.1038/s41591-020-0820-9 32284615 PMC7095063

[7] Phua J, Weng L, Ling L, et al Intensive care management of coronavirus disease 2019 (COVID-19): challenges and recommendations. Lancet Respir Med 2020;8:506–17. 10.1016/S2213-2600(20)30161-2 32272080 PMC7198848

[8] Bourouiba L Turbulent gas clouds and respiratory pathogen emissions: potential implications for reducing transmission of COVID-19. JAMA 2020;323:1837–8. 10.1001/jama.2020.4756 32215590

[9] Park M, Cook AR, Lim JT, et al A systematic review of COVID-19 epidemiology based on current evidence. J Clin Med 2020;9:967 10.3390/jcm9040967 PMC7231098 32244365

[10] Okba NM, Muller MA, Li W, et al SARS-CoV-2 specific antibody responses in COVID-19 patients. medRxiv 2020;26:1478–88. 10.1101/2020.03.18.20038059 PMC7323511 32267220

[11] Mather C Avian influenza multiple: enacting realities and dealing with policies in South Africa’s farmed ostrich sector. J Rural Stud 2014;33:99–106. 10.1016/j.jrurstud.2013.04.005

[12] Cheng VC-C, Wong S-C, Chuang VW-M, et al The role of community-wide wearing of face mask for control of coronavirus disease 2019 (COVID-19) epidemic due to SARS-CoV-2. J Infect 2020;81:107–14. 10.1016/j.jinf.2020.04.024 32335167 PMC7177146

[13] Gandhi M, Beyrer C, Goosby E Masks do more than protect others during COVID-19: reducing the inoculum of SARS-CoV-2 to protect the wearer. J Gen Intern Med 2020;35:1–4. 10.1007/s11606-020-06067-8 PMC7393808 32737790

[14] McKee M, Stuckler D If the world fails to protect the economy, COVID-19 will damage health not just now but also in the future. Nat Med 2020;26:640–2. 10.1038/s41591-020-0863-y 32273610

[15] Enserink M, Kupferschmidt K With COVID-19, modeling takes on life and death importance. Science 2020;367:1414.2–5. 10.1126/science.367.6485.1414-b 32217707

[16] Hinchliffe S Model evidence–the COVID-19 case. Somatosphere 2020 http://somatosphere.net/forumpost/model-evidence-covid-19/

[17] Van Damme W, Dahake R, Delamou A, et al The COVID-19 pandemic: diverse contexts; different epidemics-how and why? BMJ Glob Health 2020;5:e003098. 10.1136/bmjgh-2020-003098 PMC7392634 32718950

[18] Giles-Vernick T, Kutalek R, Napier D, et al A new social sciences network for infectious threats. Lancet Infect Dis 2019;19:461–3. 10.1016/S1473-3099(19)30159-8 31034383

[19] Ashraf H, Mol A Outsides and insides: Covid-19 seen from the first floor of a house in Mirpur, Dhaka. Somatosphere 2020 http://somatosphere.net/2020/outsides-insides-covid-19-mirpur.html/

[20] Narasimhan M, Allotey P, Hardon A Self care interventions to advance health and wellbeing: a conceptual framework to inform normative guidance. BMJ 2019;365:l688. 10.1136/bmj.l688 30936087 PMC6441866

